# Supplementary material for: Multi-View Spatial-Temporal Graph Convolutional Networks With Domain Generalization for Sleep Stage Classification
Source: IEEE Trans Neural Syst Rehabil Eng. Author manuscript; Available in PMC 2021 Oct 30. (PMC8556658; doi:10.1109/TNSRE.2021.3110665)
Supplement: supp1-3110665 [file NIHMS1744869-supplement-supp1-3110665.pdf]

Supplementary Material

S.1 The detailed construction process of two brain view graphs

Graphs are used to describe the connections among brain regions and features of each channel. As we all know, graph consists of nodes and edges. In this work, we regard each EEG channel as a node and the connections among brain regions as an edge. Figure S.1 describes the construction process of functional connectivity-based brain graph G^{FC} and spatial distance-based brain graph G^{DC} in more detail. Each node has its own attributes (features). To extract the features, two network branches with different sizes of convolution kernels (32 and 64) is employed to generate the feature matrix from raw EEG (from the upper-left box to the lower-left box). Functional connectivity (FC) graph and distance-based connectivity (DC) graphs share the same node features, but have different edges. Functional connectivity is generated based on the feature matrix, and distance-based connectivity is generated according to the relative position and distance of EEG electrodes on the head. As the lower-right box in Figure S.1 shows, functional connectivity and distance-based connectivity form the DC graph and FC graph, respectively.

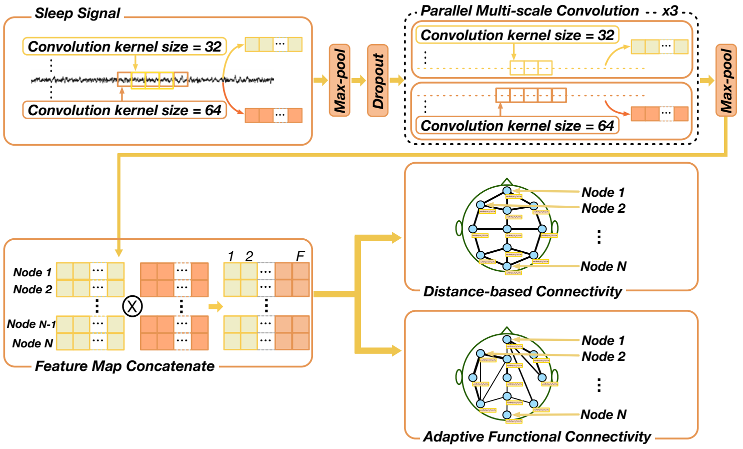

Figure S.1: The detailed construction process of functional connectivity-based brain graph and spatial distance-based brain graph.

Table S.1: Feature extraction network topology, which is a two branch CNN. Small convolution kernel is better at capturing temporal information, while large convolution kernel is better at capturing frequency information.

	Output dim	Filters	Kernel	Stride	Activation	Pad	Output dim	Filters	Kernel	Stride	Activation	Pad
Input	3000×1											
Conv1D+BN	492×32	32	50	6	ReLU	valid	53×64	64	400	50	ReLU	valid
MaxPool1D	30×32	-	16	16	-	valid	6×64	-	8	8	-	valid
Dropout	30×32	-	0.5	-	-	-	6×64	-	-	-	-	-
Conv1D+BN	30×64	64	8	1	ReLU	same	6×64	64	6	1	ReLU	same
Conv1D+BN	30×64	64	8	1	ReLU	same	6×64	64	6	1	ReLU	same
Conv1D+BN	30×64	64	8	1	ReLU	same	6×64	64	6	1	ReLU	same
MaxPool1D	3×64	-	8	8	-	valid	1×64	-	4	4	-	valid
Flatten	192	-	-	-	-	-	64	-	-	-	-	-
Concatenate	256											

S.2 Dataset statistics

Table S.2: Number of 30s epochs for each sleep stage on ISRUC-S3 dataset

	Wake	N1	N2	N3	REM	Total
Sample Number	1651	1215	2609	2014	1060	8549
Proportion	19.3%	14.2%	30.5%	23.6%	12.4%	100%

Table S.3: Number of 30s epochs for each sleep stage on MASS-SS3 dataset

	Wake	N1	N2	N3	REM	Total
Sample Number	6277	4819	29749	7651	10560	59056
Proportion	10.6%	8.2%	50.4%	13.0%	17.9%	100%

S.3 Baseline Methods

We compare the proposed model with the following baselines:

- **Ref[2]**: Support vector machine is a typical machine learning method for classification tasks.
- **Ref[3]**: Random forests are an ensemble learning method for classification tasks.
- **Ref[11]**: A mixed neural network, which combines multilayer perceptron (MLP) and LSTM.
- **Ref[12]**: A mixed model combines CNN and BiLSTM to capture time-invariant features and transition rules among sleep stages.
- **Ref[7]**: A temporal sleep stage classification apply multivariate and multimodal time series.
- **Ref[13]**: SeqSleepNet changes the single sleep stage classification problem into a sequence-to-sequence classification problem by using attention-based bidirectional RNN (ARNN) and RNN.
- **Ref[15]**: A spatial-temporal graph neural network with the adaptive sleep graph learning mechanism achieves the state-of-the-art performance.

S.4 Confusion matrices

Figure S.2 and Figure S.3 respectively present the confusion matrix of MSTGCN on different datasets.

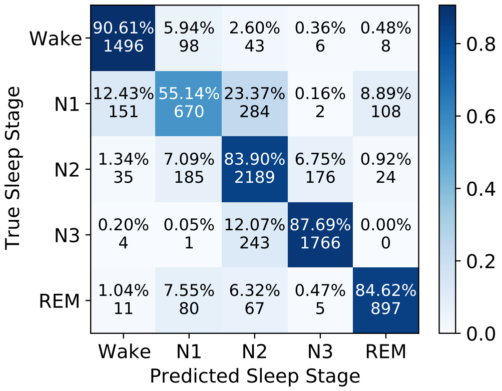

Figure S.2: The confusion matrix of MSTGCN on ISRUC-S3 dataset.

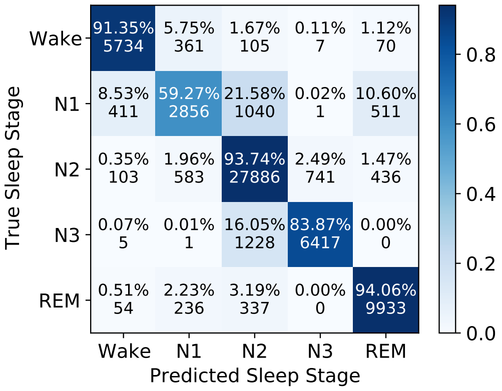

Figure S.3: The confusion matrix of MSTGCN on MASS-SS3 dataset.

S.5 Comparison of hypnograms scored by MSTGCN and sleep experts

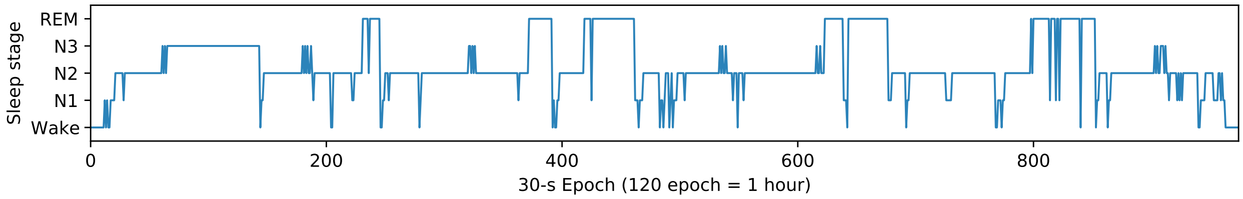

(a) Hypnogram manually scored by sleep experts.

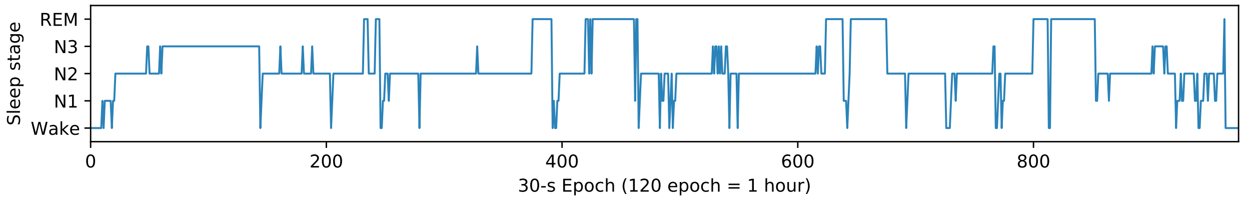

(b) Hypnogram automatically scored by the proposed MSTGCN.

Figure S.4: Output hypnogram produced by the proposed MSTGCN compared to the ground-truth. The hypnograms present that the proposed MSTGCN is able to classify most of the sleep stages correctly.

S.6 Effect of Different Network Configurations

To further investigate how hyper-parameter settings affect the model performance, we conduct experiments with different network configurations. The same experiment hyperparameter setting is utilized in all experiments except the studied varying factor. As shown in Figure S.5, the proposed MSTGCN is not sensitive to the hyper-parameter settings. Fewer ST-GCN layers and more convolution kernels help to improve performance slightly. The appropriate Chebyshev polynomial K and regularization parameter λ is also conducive to the improvement of the model performance.

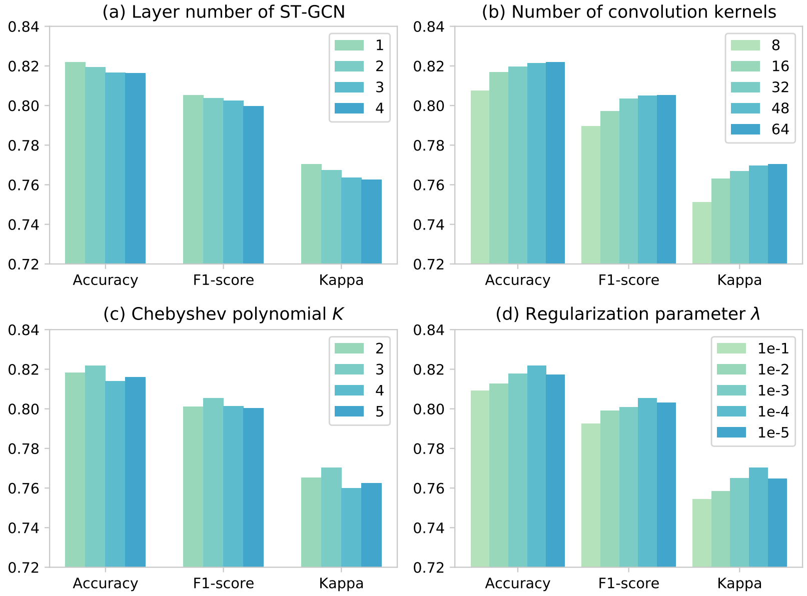

Figure S.5: Network configuration analysis. The four figures respectively show the effect of layer number of ST-GCN, number of convolution kernels, Chebyshev polynomial K, and regularization parameter λ.
